# Transcript analysis of laser capture microdissected white matter astrocytes and higher phenol sulfotransferase 1A1 expression during autoimmune neuroinflammation

**DOI:** 10.1186/s12974-015-0348-y

**Published:** 2015-07-04

**Authors:** Flora Guillot, Alexandra Garcia, Marion Salou, Sophie Brouard, David A. Laplaud, Arnaud B. Nicot

**Affiliations:** INSERM UMR 1064, CHU Hôtel-Dieu, 30 Bvd Jean Monnet, 44093 Nantes, France; Université de Nantes, Faculté de Médecine, Nantes, France; CESTI/ITUN, CHU de Nantes, Nantes, France; Service de Neurologie, CHU de Nantes, Nantes, France

**Keywords:** Multiple sclerosis, Fibrous astrocyte, Radial glia, Astrogliosis, Estrogen metabolism

## Abstract

**Background:**

Astrocytes, the most abundant cell population in mammal central nervous system (CNS), contribute to a variety of functions including homeostasis, metabolism, synapse formation, and myelin maintenance. White matter (WM) reactive astrocytes are important players in amplifying autoimmune demyelination and may exhibit different changes in transcriptome profiles and cell function in a disease-context dependent manner. However, their transcriptomic profile has not yet been defined because they are difficult to purify, compared to gray matter astrocytes. Here, we isolated WM astrocytes by laser capture microdissection (LCM) in a murine model of multiple sclerosis to better define their molecular profile focusing on selected genes related to inflammation. Based on previous data indicating anti-inflammatory effects of estrogen only at high nanomolar doses, we also examined mRNA expression for enzymes involved in steroid inactivation.

**Methods:**

Experimental autoimmune encephalomyelitis (EAE) was induced in female C57BL6 mice with MOG_35–55_ immunization. Fluorescence activated cell sorting (FACS) analysis of a portion of individual spinal cords at peak disease was used to assess the composition of immune cell infiltrates. Using custom Taqman low-density-array (TLDA), we analyzed mRNA expression of 40 selected genes from immuno-labeled laser-microdissected WM astrocytes from lumbar spinal cord sections of EAE and control mice. Immunohistochemistry and double immunofluorescence on control and EAE mouse spinal cord sections were used to confirm protein expression in astrocytes.

**Results:**

The spinal cords of EAE mice were infiltrated mostly by effector/memory T CD4+ cells and macrophages. TLDA-based profiling of LCM-astrocytes identified EAE-induced gene expression of cytokines and chemokines as well as inflammatory mediators recently described in gray matter reactive astrocytes in other murine CNS disease models. Strikingly, SULT1A1, but not other members of the sulfotransferase family, was expressed in WM spinal cord astrocytes. Moreover, its expression was further increased in EAE. Immunohistochemistry on spinal cord tissues confirmed preferential expression of this enzyme in WM astrocytic processes but not in gray matter astrocytes.

**Conclusions:**

We described here for the first time the mRNA expression of several genes in WM astrocytes in a mouse model of multiple sclerosis. Besides expected pro-inflammatory chemokines and specific inflammatory mediators increased during EAE, we evidenced relative high astrocytic expression of the cytoplasmic enzyme SULT1A1. As the sulfonation activity of SULT1A1 inactivates estradiol among other phenolic substrates, its high astrocytic expression may account for the relative resistance of this cell population to the anti-neuroinflammatory effects of estradiol. Blocking the activity of this enzyme during neuroinflammation may thus help the injured CNS to maintain the anti-inflammatory activity of endogenous estrogens or limit the dose of estrogen co-regimens for therapeutical purposes.

**Electronic supplementary material:**

The online version of this article (doi:10.1186/s12974-015-0348-y) contains supplementary material, which is available to authorized users.

## Background

Astrocytes are situated in key positions between microvessels, axons, and oligodendrocytes where they participate in a wide range of functions during brain construction and maintenance, exchanging signals with the neuronal compartment at the synapse, maintaining low extracellular glutamate level preventing chronic glutamate excitotoxicity, and supplying substrates for energy metabolism to neurons and oligodendrocytes [1]. Astrocytes also have the potential to secrete a variety of signaling molecules, including growth factors for neurons and oligodendrocytes, immune modulators, metalloproteases, and nitric oxide depending on cellular context. Injury, inflammation, or degenerative disease in the central nervous system (CNS) is accompanied by alterations in the morphology of astrocytes, a response referred to as reactive astrogliosis. The positive effect of the astrogliotic response was initially illustrated using mice in which early reactive proliferative astrocytes were selectively targeted for ablation in the injured CNS [2]. On the other hand, the detrimental role of reactive astrocytes involving NFkB signaling was clearly demonstrated in in vivo models of neuroinflammation, indicating they have a key role in the local inflammatory response [3] and in remyelination [4]. Thus, in addition to axonal damage and demyelination, astrocytic activation is a central pathological feature that likely contributes to multiple sclerosis progression, in relapsing-remitting as well as in progressive forms with low grade inflammation [5]. Previous studies using transcriptomics of experimental autoimmune encephalomyelitis (EAE) and multiple sclerosis (MS) lesions have provided important insights in the pathogenesis and potential new targets for therapy [6–8]. Comparing the transcriptomes of whole CNS tissues is a useful strategy for pinpointing specific alterations in CNS diseases. However, this approach needs to be completed by histological approaches to localize the cellular source of identified molecules and their potential targets. Thus, one factor limiting the interpretation of tissue-based transcriptomic data is the lack of cell-type purity. Laser capture microdissection (LCM) following immunolabeling is useful for overcoming this limitation [9]. Direct mRNA profiling for a single cell population from frozen brain tissue slices is now possible. Here, we applied this strategy to characterize the molecular profile of white matter astrocytes in EAE spinal cord. This approach is especially needed for this astrocyte population because it cannot be purified by flow cytometry-based methods in contrast to cortical gray matter astrocytes [10]. Indeed, the use of astrocyte-specific GFP transgenic mice and Fluorescence activated cell sorting (FACS) analysis has facilitated gene expression profiling of cortical astrocytes in two animal models of CNS injury indicating that astrocytes may exhibit two types of reactive profile (deleterious or protective) depending on the disease models [11]. Similarly, a better identification of the molecular signature of reactive white matter astrocytes in CNS tissue infiltrated by immune cells is a prerequisite for further designing in vitro and in vivo experiments in order to better understand the contribution of astrocytes in multiple sclerosis physiopathology. Using a murine model of multiple sclerosis, experimental autoimmune encephalomyelitis, we employed LCM-astrocytes from spinal cord fresh-frozen sections to determine transcript expression levels of various genes that have been recently highlighted in normal [10] or reactive [11] gray matter astrocytes. Whereas low levels of endogenous estrogen prevent microglial reactivity [12], we and others have found that only high nanomolar levels of estrogen are able to dampen ongoing EAE and neuroinflammation in vivo as well as in vitro to reduce astrocytic pro-inflammatory cytokines or chemokines in vitro [13]. We thus also examined the expression of sulfotransferases and glucuronidases, cytoplasmic enzymes involved in estrogen inactivation by conjugation [14].

## Materials and methods

### Induction of active EAE

Three adult female mice (8 weeks old, Janvier Labs, France) were immunized subcutaneous (s.c.) at the base of the tail and lower flanks with 200 μg of MOG_35–55_ peptide (MEVGWYRSPFSRVVHLYRNGK, purity >85 %, Genecust Europe, France) in phosphate buffered saline (PBS) emulsified with an equal part of complete Freund’s adjuvant (CFA) supplemented with Mycobacterium tuberculosis H37Ra at 6 mg/ml (Difco Laboratories, Detroit, MI, USA) as previously described [15]. Three control mice were s.c. injected with adjuvant/PBS. Mice received 300 ng of pertussis toxin injected intraperitoneally (i.p.) on days 0 and 2 (Calbiochem, Darmstadt, Germany). Mice were scored daily for clinical signs of disease according to the 0–5 scale: 0, no detectable sign; 1, complete limp tail; 2, limp tail with unilateral hindlimb paralysis; 3, bilateral hindlimb paralysis; 4, bilateral hindlimb paralysis and forelimb weakness (end point). At peak of disease (score 3–4, 5–6 days after EAE onset corresponding to partial limp tail), mice were anesthetized with thiopental and intracardiacally perfused with cold PBS. Spinal cords were flushed. The lumbar part was snap-frozen and embedded in Tissue-Tek® optimal cutting temperature (OCT) with dry ice and stored at −80 °C and placed at −20/25 °C before being used for LCM. The rest of the spinal cord was used for FACS analysis.

### Assessment of spinal cord immune infiltrate by FACS analysis

Immune cell infiltrates were evaluated in individual spinal cords according to the method adapted from [16]. Spinal cord was dilacerated and digested for 20 min in HBBS buffer containing collagenase D (1 mg/ml), DNase I (1 mg/ml), and MgCl_2_ (1 mM). Cells were dissociated and passed through 100 μm and 70 μm filters. Cells were then incubated with fluorescent antibody anti-CD45 APC-Cy7 (BD 560694), anti-CD11b APC (BD 553312), anti-CD3 FITC (BD 553062), anti-CD4 A700 (BD 557956), anti-CD8 PE-Cy7 (BD 552877), anti-B220 PE (BD 553090) or anti-Ly6G PE (BD 551461), anti-CD44 A700 (BD 560567), and anti-CD62L PerCP-Cy5.5 (BD 560513) at 1/100 for 15 min at 4 °C. Next, cells were permeabilized with BD CytoFix/CytoPerm (554714, Biosciences), and nuclei were labeled with DAPI (1 μg/ml) before gating on DAPI+ immune cells (CD45high) for FACS analysis. Table 1 recapitulates antibodies and their cellular targets used for this study.

**Table 1:** List of antibodies used for FACS analysis with their corresponding targets

Antibody	Targets
Anti-CD45.2	Immune cells (high level) and microglia (intermediate level)
Anti-CD11b	Granulocytes/neutrophiles, monocytes/macrophages, microglia
Anti-CD3	T cells (with anti-CD4 for CD4 T cells; with anti-CD8 for CD8 T cells)
Anti-B220	Cells of the B cell lineage
Anti-CD19	Cells of the B cell lineage (high level before plasma blast stage)
Anti-CD44	Memory/effector T cells
Anti-CD62L	Naive (CD44^−^) and central memory (CD44^+^) T cells
Anti-Ly6G	Neutrophiles

### Preparation and staining of frozen sections for LCM

Twelve micrometer spinal cord sections were prepared using a Leica cryostat at −17 °C and thawed onto glass slides covered with a thin biochemically inert membrane (MembraneSlide 0.17 PET, Zeiss, Germany). After quick drying at room temperature, the sections were fixed in 100 % ethanol, blocked for 5 min at room temperature in 0.01 M phosphate buffer containing 2 M NaCl (high PBS), 25 % Normal Donkey Serum (NDS), and 0.1 % Triton-X100. Sections were then incubated with primary antibodies rabbit anti-glial fibrillary acidic protein (GFAP) (1/50, DakoCytomation, Glostrup, Denmark) and rat anti-mouse CD3e (1/10, BD Pharmingen) in high PBS with 2.5 % NDS for 10 min. After two 3-min washings with high PBS, sections were incubated with secondary antibodies (1/50) in high PBS and 2.5 % NDS for 10 min. The AF488-coupled F(ab′)2 secondary (anti-rabbit IgG) and Rhodamine Red-X coupled F(ab′)2 secondary (anti-rat IgG) from Jackson ImmunoResearch (Suffolk, England) were used. After two 3-min washings with high PBS, sections were dipped in 70 % ethanol for 1–3 s and dehydrated in 100 % ethanol.

### Laser capture microdissection and RNA extraction

**Fig. 1:**
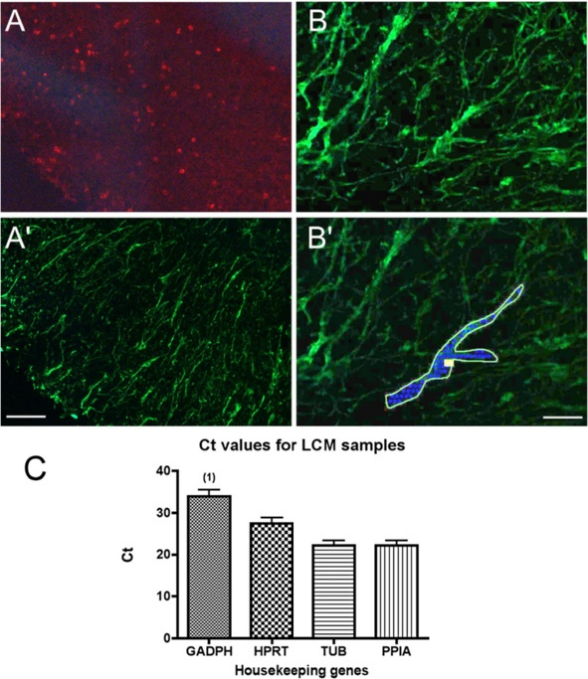
Immunolabeled LCM-astrocytes and housekeeping gene expression. **a**, **b** Example of immunostainings of T cells and astrocytes in white matter spinal cord (here ventrolateral part as in Fig. 3). CD3 was used to label T cells and identify immune T cell infiltrated zones (**a**) and GFAP to label astrocytes (**a′**, **b**, **b′**). **b′** shows an example of an astrocyte drawing before cutting and catapulting. Scale bar **a**, **a′** 70 μm; **b**, **b′**, 25 μm. **c** Analysis of the four housekeeping genes expressed in the LCM-dissected astrocyte control and EAE samples (*n* = 6, no differences between control and EAE samples were observed except that one of six cDNA samples did not give a signal for GADPH (1)). TUB1A1 (TUB) and PPIA probes gave the most robust signals; the geometric mean of these two reference genes was thus used for dCt calculations

PET-coated slides were placed face up, dried directly after staining, and a 0.2-ml Eppendorf tube cap was placed on the dedicated tubing rack of the UV laser microdissection PALM Microbeam (Carl Zeiss, Germany). Samples were processed within 2 h with LCM settings as follows: cut energy, 41; cut focus, 82; LPC energy delta, LPC focus delta 2; and 20 μm spot distances. After stamping CD3-immunolabeled cells at ×40 objective under Zeiss 43HE filter set to localize T cell infiltrated zones in the EAE white matter, contours of GFAP-labeled astrocytes were carefully drawn at ×40 objective using Zeiss 38HE filter set (Fig. 1a, b). White matter GFAP-labeled astrocytes from control mice were directly selected under the 38HE filter set. At the end of the drawings, astrocytes were microdissected and catapulted in the 0.2-ml Eppendorf tube cap filled with RLT lysis buffer (RNeasy Micro kit, Qiagen, Hilden, Germany) containing 1 % beta-mercaptoethanol. For each mouse, ~5.10^6^ μm^2^ of pooled astrocytes were then extracted for total RNA and Dnase I treated with RNeasy Micro kit. RNA quantity and integrity were evaluated using the 2100-Bioanalyzer with the RNA 6000 Pico kit (Agilent Technologies). Astrocyte samples (corresponding to 4000–6000 cells) gave 5–7 ng RNA. To analyze more than a few genes, a preamplification step was thus required. Taqman low-density-arrays (TLDAs) after preamplification of LCM samples have been shown to be highly sensitive and reproducible [17]. The cDNA was amplified with the CellAmp Whole Transcriptome Amplification Kit (#TAK3734Z, Takara, Japan).

### Taqman arrays

Custom-made Taqman array 96 well plates (Life Technologies, Foster City, CA, USA) were used for gene expression profiling based on qPCR to compare EAE reactive astrocytes to control astrocytes. The list of genes and corresponding probes is presented in Table 2. The Taqman array plates were loaded with the sample-specific PCR mix containing the template cDNA, TaqMan Universal PCR Master Mix, and water to form the reaction mix as indicated by the provider. Then, the plates were centrifuged with two consecutive 1-min spins to ensure complete distribution of the sample-specific PCR reaction mix. The plate was sealed and introduced into Viia7 Real-Time PCR system (Applied Biosystems/Life Technologies, Foster City, CA, USA) with 40 PCR cycles and according to recommended thermal cycling conditions for microfluidic cards. To detect expression signals at the maximum sensitivity, cycle threshold (Ct) readings were obtained using a DRn threshold of 0.04, ensuring this was in the log-linear range of exponential amplification for each gene and at least 10-fold above background levels. We rated a Ct value of <38 as negative (below detection level), 38–36 as very poor, 35–30 as poor, and <30 as good. The relative expression of each transcript was measured using the 2^−ddCt^ method [18]; the geometric mean of Ct values of the best reference genes (according to [19]), alpha-tubulin 1A (TUB1A1) and peptidylprolyl isomerase A (PPIA, cyclophilin A) (Fig. 1c), was used for normalization of gene expression levels and calculation of dCt values. Values are expressed as mean ± sem. Data were analyzed using Excel and GraphPad Prism software 5.0 using Mann–Whitney test. Differences were considered statistically significant if the *P* value was <0.05.

**Table 2:** List of gene transcripts with their corresponding probes (ID assay)

Symbol	ID assay	Gene name
Arg1	Mm00475988_m1	Arginase 1
Ccl2	Mm00441242_m1	Chemokine (C-C motif) ligand 2
Ccl3	Mm00441259_g1	Chemokine (C-C motif) ligand 3
Ccl5	Mm01302427_m1	Chemokine (C-C motif) ligand 5
Ccl7	Mm00443113_m1	Chemokine (C-C motif) ligand 7
Cxcl9	Mm00434946_m1	Chemokine (C-X-C motif) ligand 9
Cxcl10	Mm00445235_m1	Chemokine (C-X-C motif) ligand 10
Cxcl12	Mm00445553_m1	Chemokine (C-X-C motif) ligand 12
Cxcl16	Mm00469712_m1	Chemokine (C-X-C motif) ligand 16
Dbi	Mm00833261_g1	Diazepam binding inhibitor
Gfap	Mm01253033_m1	Glial fibrillary acidic protein
Il15	Mm00434210_m1	Interleukin 15
Il15ra	Mm04336046_m1	Interleukin 15 receptor, alpha chain
Il1b	Mm00434228_m1	Interleukin 1 beta
Lcn2	Mm01324470_m1	Lipocalin 2
Mbp	Mm01266402_m1	Myelin basic protein
Serping1	Mm00437834_m1	Serpin peptidase inhibitor, clade G, member 1
Spp1	Mm00436767_m1	Secreted phosphoprotein 1 (osteopontin)
Sult1a1	Mm00467072_m1	Sulfotransferase family 1A, phenol-preferring, member 1
Sult1c2	Mm00471849_m1	Sulfotransferase family, cytosolic, 1C, member 2
Sult1d1	Mm00502035_m1	Sulfotransferase family 1D, member 1
Sult1e1	Mm00499178_m1	Sulfotransferase family 1E, member 1
Sult2a1	Mm04205659_mH	Sulfotransferase family 2A, DHEA-preferring, member 2
Sult2b1	Mm00450550_m1	Sulfotransferase family, cytosolic, 2B, member 1
Sult3a1	Mm00491057_m1	Sulfotransferase family 3A, member 1
Tgfb1	Mm01178820_m1	Transforming growth factor, beta 1
Tnf	Mm00443258_m1	Tumor necrosis factor alpha
Tnfrsf9	Mm00441899_m1	Tumor necrosis factor receptor superfamily, member 9
Tnfsf9	Mm00437155_m1	Tumor necrosis factor (ligand) superfamily, member 9
Tspo	Mm00437828_m1	Translocator protein
Ugt1a1	Mm02603337_m1	UDP glucuronosyltransferase 1 family, polypeptide A1
Ugt2a3	Mm00472170_m1	UDP glucuronosyltransferase 2 family, polypeptide A3
Ugt2b34	Mm00655373_m1	UDP glucuronosyltransferase 2 family, polypeptide B34
Ugt2b5	Mm01623253_s1	UDP glucuronosyltransferase 2 family, polypeptide B5
Ugt3a1	Mm01703504_mH	UDP glycosyltransferases 3 family, polypeptide A1
Ugt8a	Mm00495930_m1	UDP galactosyltransferase 8A
Housekeeping genes		
Hprt	Mm00446968_m1	Hypoxanthine guanine phosphoribosyl transferase
Ppia	Mm02342430_g1	Peptidylprolyl isomerase A
Tuba1a	Mm00846967_g1	Tubulin, alpha 1A
Gadph	Mm99999915_g1	Glyceraldehyde-3-phosphate dehydrogenase

### Immunocytochemistry on spinal cord sections

For double immunofluorescence, fresh-frozen spinal cord 12-μm thick sections from control and EAE (score 3) mice were alcohol fixed and preincubated in PBS with 25 % Normal Donkey Serum and 0.03 % Triton-X100 for 20 min at room temperature, followed by overnight incubation at 4 °C with rat anti-GFAP (1/400, #345860, Merck Millipore) and rabbit anti-mouse SULT1A1 (1/400, #38411, Abcam) in PBS and 2.5 % NDS and 0.03 % Triton-X100. After three washes in PBS, sections were incubated for 45 min with donkey AF594- or AF488-coupled F(ab′)2 anti-rat or anti-rabbit IgG (1:1500; Jackson ImmunoResearch, Suffolk, England) in PBS, 2.5 % NDS and 0.03 % Triton-X100. Sections were incubated with DAPI at 1 μg/ml for 5 min, rinsed, and coverslipped with anti-fading mounting medium (Mowiol/DABCO). Negative controls, where anti-SULT1A1 or anti-SULT1A1/anti-GFAP was omitted in the incubation steps, were included in the experiment. The spinal cord sections were analyzed on a fluorescent microscope using appropriate filters (Nikon), and pictures were taken at ×40 objective using a digital camera (Eclipse DXM1200) connected to an image-acquisition software (ACT-1, Nikon).

For DAB colorimetric immunohistochemistry, coronal spinal cord 16-μm thick sections from paraformaldehyde-perfused control (3) and EAE (3) mice (28 days after EAE onset, score 1.5) were used for SULT1A1 and GFAP immunolabeling using peroxydase/DAB amplification. Briefly, sections were rehydrated in 0.01 M PBS, incubated with 0.3 % H202 for 20 min, rinsed in PBS, and preincubated in PBS with 25 % Normal Donkey Serum (NDS) and 0.03 % Triton-X100 for 20 min at room temperature, followed by overnight incubation at 4 °C with anti-mouse SULT1A1 rabbit antibody at 1/1000 (Bioss, Woburn, MA, USA) or at 1/400 (Abcam, #38411, Cambridge, MA, USA) or with rabbit anti-GFAP (1/2000, Dako) in PBS and 2.5 % NDS and 0.03 % Triton-X100. After three washes in PBS, sections were incubated with anti-rabbit IgG donkey biotinylated-IgG (Jackson ImmunoResearch, Suffolk, England) for 45 min followed by three washes in PBS. Finally, peroxydase revelation was performed using ABC Elite kit (Vector Labs, Burlingame, CA, USA) and using 10-min DAB substrate incubation at room temperature. Sections were then dehydrated and coverslipped with Neo-Mount reagent (Merck Millipore, Darmstadt, Germany). As control for SULT1A1 specificity, negative controls with no primary antibody performed on spinal cord and kidney sections were performed and did not give DAB staining. Female mouse kidney which contains SULT1A1-expressing tubular cells was used as positive control for SULT1A1 antibodies. Pictures were taken at ×40 objective using a digital camera (Eclipse DXM1200) connected to an image-acquisition software (ACT-1, Nikon).

## Results and discussion

### FACS analysis of EAE infiltrates

A portion of the spinal cord from the three EAE mice used for LCM was processed for FACS analysis of immune infiltrates. The leukocyte composition is in agreement with previous reports with 33 ± 8 % lymphocytes (CD11b-), 39 ± 14 % macrophages (CD11b + Ly6G-), and 13 ± 9 % neutrophiles (CD11b + Ly6G+). Lymphocytes were composed of 64 ± 4 % T cells (CD3+) with a CD4/CD8 ratio of ~4 and 23 ± 4 % B cell lineage (B220+); more than 80 % of T cells were CD44+ CD62L- indicating an effector/memory phenotype (Additional file 1: Figure S1).

### RNA is well preserved after the different immunolabeling and LCM steps

**Fig. 2:**
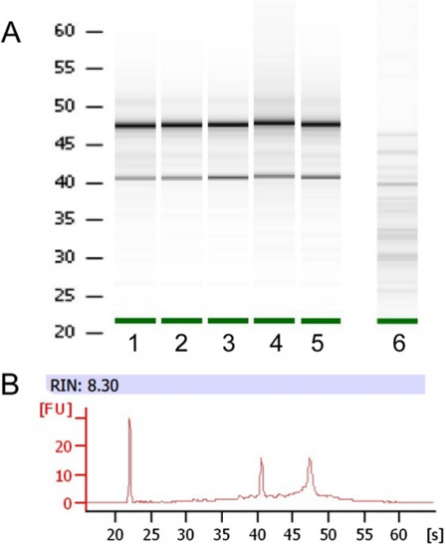
Assessment of RNA integrity during immunohistochemical steps and laser microdissection procedures. **a** Microelectrophoresis of total RNA extracted from 8–12 coronal fresh-frozen spinal cord sections: (1) directly after quick defrosting (starting material), (2) after ethanol fixation, (3) at the end of the LCM immunohistochemistry steps in the presence of high NaCl concentration (high PBS), (4) with further 2 h dry at room temperature corresponding to the maximum time needed for drawing, dissecting, and capturing astrocytes on one slide, (5) after the microdissection of astrocytes, and (6) at the end of immunohistochemistry steps in the presence of isotonic NaCl concentration (normal PBS). **b** Example of an electrophoregram with RIN value corresponding to (5)

In order to ensure that the immunostaining or microdissection steps preserved sufficient RNA, we first checked the integrity of the RNA extracted from the whole spinal cord coronal sections at each critical point of the protocol. Automatic analysis of the 28S/18S ratio using Agilent microelectrophoresis indicates a good preservation of RNAs (ratios >1.6) with our LCM protocol (Fig. 2a, lanes 1–5). The use of high (2 M) NaCl concentration in all immunolabeling steps was crucial to prevent loss and degradation of the RNA (Fig. 2a, lane 6) as already mentioned [20]. The RNA integrity number (RIN) was superior to 6.7 for the samples (example of a microelectrophoresis profile in Fig. 2b) indicating relative well-preserved RNA, whereas it dropped to 4–4.6 when high NaCl was omitted in the incubation steps. RINs for the six LCM samples used for TDLA were 7.4 ± 0.3, indicating good RNA quality.

### TLDA analysis

We present here a selected transcriptomic profile of 40 genes of control and EAE spinal cord white matter astrocytes. About 4000–6000 pooled LCM-astrocytes per sample (corresponding to a total captured surface of 5 mm^2^) were needed for obtaining sufficient RNA (~5 ng). This recovery is in the expected range for LCM-dissected mouse cells (1–10 pg/cell; [21]). Table 3 summarizes the expression of transcripts that was examined in this study. All transcripts presented here were at least detected in the mixed tissue control sample, which contains liver, spleen, and brain cDNAs and which was used as a positive control for the probes.

**Table 3:** Summary of TLDA analysis

	Mix tissue	LCM-astrocytes				
	Ct value	dCt Con	dCt EAE	Fold increase (2^−ddCt^)		
Gene symbol		Detected in all EAE samples at poor to good levels				
Arg1	25.1	14.3 ± 1.0	13.2 ± 1.5	2.2	NS	¥
*Ccl2*	23.3	12.0 ± 0.5	3.5 ± 0.6	*349*	*	¥
Ccl3	23.9	11.1 ± 2.7	11.8 ± 4.4	0.6	NS	
*Ccl5*	18.5	12.0 ± 2.5	4.4 ± 1.1	*199*	*	¥
*Ccl7*	23.9	12.5 ± 2.1	4.6 ± 0.4	*231*	*	¥
*Cxcl9*	21.3	13.0 ± 1.7	4.9 ± 0.9	*270*	*	¥
*Cxcl10*	21.7	13.8 ± 1.1	6.1 ± 0.6	*214*	*	¥
*Cxcl16*	21.4	9.8 ± 1.9	5.0 ± 0.5	*28*	*	
*Dbi*	12.8	0.7 ± 0.2	−0.2 ± 0.2	*1.8*	*	
Gfap	24.7	9.7 ± 1.2	11.9 ± 3.0	0.2	NS	
*Lcn2*	13.7	10.5 ± 2.9	2.5 ± 0.4	*253*	*	
Mbp	22.1	7.9 ± 1.7	7.4 ± 1.5	1.4	NS	
*Serping1*	16.5	11.0 ± 2.5	5.4 ± 0.3	*48*	*	
*Spp1*	23.2	13.1 ± 1.1	7.3 ± 1.0	*54*	*	
*Sult1a1*	18.1	10.5 ± 0.5	7.5 ± 0.7	*7.7*	*	
Tnf	33.3	10.2 ± 0.8	13.9 ± 1.5	0.1	NS	
*Tspo*	17.6	10.7 ± 2.8	5.4 ± 1.1	*31*	*	
		Detected at poor levels in some EAE samples				
Il1b	26.2	Detected only in one EAE sample				
Il15	23.4	Detected only in two EAE samples				
Il15ra	22.9	Detected only in two EAE samples				
Tnfrsf9	28.8	Detected only in one EAE sample				
Tnfsf9	29.3	Detected only in one EAE sample				
		Detected at very poor levels in all samples				
Cxcl12	24.6	13.9 ± 0.8	14.6 ± 1.7			
Tgfb1	33.6	13.9 ± 1.1	18.2 ± 0.5			
Ugt8a	30.1	12.7 ± 1.7	17.7 ± 0.4			
		Undetected in all samples				
Sult1c2	25.0		Undetected			
Sult1d1	22.8		Undetected			
Sult1e1	25.1		Undetected			
Sult2a1	18.3		Undetected			
Sult2b1	23.2		Undetected			
Sult3a1	19.7		Undetected			
Ugt1a1	24.2		Undetected			
Ugt2a3	21.6		Undetected			
Ugt2b5	20.2		Undetected			
Ugt2b34	23.3		Undetected			
Ugt3a1	23.3		Undetected			

Ct value for mixed tissue which contained liver, spleen, and brain cDNA indicated that the probes were effective in detecting the transcript. dCt values for control and EAE correspond to the Ct value of the target gene minus the Ct geometric mean of the two housekeeping genes. For LCM-astrocyte samples, dCt values are expressed as mean ± sem. Transcripts showing significant EAE-induced increased expression are in italics

*NS p* > 0.05, *¥* undetected in two of the three control samples (Ct value fixed to 38)

*p < 0.05

### Glial markers

Expression of the astrocytic marker GFAP mRNA was detected in all LCM samples although at fair levels with no difference between control and EAE astrocytes. A slight increase of almost two-fold in GFAP mRNA is found in the spinal cord tissue of various ascending EAE models; this increase can be partly explained by an increased number of astrocytes in the white matter at EAE onset and increased GFAP expression in gray matter astrocytes, which express lower levels of GFAP than white matter astrocytes in basal conditions. Moreover, GFAPα (the main GFAP isoform in white matter astrocytes) mRNAs are localized all along the processes in contrast to most mRNA concentrated around the nucleus [22]. Because LCM targeted proximal processes, it is possible that GFAPα mRNA is underrepresented in our samples, accounting for the lack of a detectable GFAP transcript increase during EAE. Fair levels of MBP mRNA, specific for oligodendrocytes, were obtained, whereas UGT8a transcripts, also specific for the oligodendroglial population [10], were detected either at very poor levels or not at all. MBP mRNA, one of the most abundant transcripts in the CNS, was also recovered from mouse LCM-astrocytes in a similar study [23], suggesting that this abundant mRNA present in nearby oligodendrocyte processes is easily amplified even from minute amounts of selected astrocytes in situ.

### Markers of astrocyte reactivity

Lcn2 though not specific to the astrocytic population is now considered a good marker of reactive astrocytes because its expression is highly induced in various experimental settings and CNS diseases including EAE [24–27]. It was thus used as a prototypic marker of astrocyte reactivity. Indeed, we found this transcript highly up-regulated in EAE samples validating the LCM approach. We also look at arginase 1, translocator protein (TSPO) and its ligand Diazepam Binding Inhibitor (DBI), serpinG1, and osteopontin (SPP1) transcripts because they have been highlighted as differentially induced in other models of neuroinflammation, particularly low induction of arginase 1 with high induction of TSPO, and serpinG1 may be associated to the pro-inflammatory profile of LPS-reactive astrocytes; in contrast, high induction of Arg1 transcripts with moderate increase in serpinG1 has been associated to a protective astrocytic profile in murine models of stroke, after transient middle cerebral artery occlusion (MCAO) [11] or photothrombotic ischemia [28]. Indeed, very high Arg1 transcripts are found in spinal cord tissue during murine EAE [29]. However, we found that Arg1 mRNA was poorly expressed in white matter astrocytes during EAE (Table 3) indicating that astrocytes are not a main source for arginase 1 production. Rather, during EAE, some activated microglia or macrophages have been shown to express high levels of arginase 1, possibly having anti-inflammatory effects [30]. TNFα is a potent pro-inflammatory cytokine for astrocytes. Although astrocytes exhibit low basal levels of this transcript, it is not up-regulated in EAE reactive astrocytes (Table 3). The increased TNFα expression in the CNS reported in previous EAE transcriptomic studies is rather ascribed to reactive microglia and infiltrated immune cells [31, 32]. Microglial TNFα is indeed highly detrimental to oligodendrocytes [33]. The interleukins IL-1β and IL-15 with its receptor IL15RA, as TNFSF9/4-1BBL and its receptor, were not consistently detected in LCM-astrocytes, though they can be expressed by cultured astrocytes in inflammatory conditions. In EAE CNS tissue, activated microglia/macrophages and infiltrating lymphocytes are in fact the main producers of IL-1β in EAE CNS tissue [34], whereas IL-15 expression is mostly endothelial and its receptor IL15RA mostly neuronal [35, 36].

Interestingly, whereas microglia was believed to be the main producer of TSPO, CNTF-stimulated GFAP+ mouse striatal astrocytes [37] and reactive astrocytes in the MCAO murine model [11] clearly overexpress the 18 kDa TSPO. The TSPO, by interacting with mitochondrial membrane proteins, is known to play a role in cholesterol mitochondrial import and thus in the first step of steroid production but also in various other functions including reactive oxygen species (ROS) generation. We found TSPO and DBI transcripts well expressed in control astrocytes and further up-regulated in LCM-reactive astrocytes (Table 3), indicating that TSPO/DBI system is part of the astrocytic reactive signature during autoimmune neuroinflammation. In line with these observations, TSPO and DBI transcript expressions have been shown to be slightly increased in MS lesions [38], but the cellular origin has not yet been described. The increased osteopontin (SPP1) expression in EAE LCM-astrocytes (Table 3) was somehow expected. We previously showed SPP1 immunoreactivity in infiltrating cells, mainly CD11c+ dendritic cells in late EAE [13], in agreement with previous reports showing osteopontin expression by macrophages/dendritic cells [39] but also by activated CCR2+ CCR5+ T cells [39] and B cells [40]. However, within the extracellular compartment of the white matter lesions, we previously observed a diffuse pattern of osteopontin immunoreactivity that could come from extracellular osteopontin secreted by immune cells and early reactive astrocytes. Here, SPP1 transcript was clearly overexpressed in reactive astrocytes at EAE peak (5–6 days post EAE onset). In line with these observations, osteopontin is also already overexpressed in the normal appearing white matter of MS brains and predominantly confined to astrocytes [41]. Finally, serpinG1 mRNA was found to be also up-regulated in EAE LCM-astrocytes. Transcript for serpinG1 (protease C1 inhibitor), a major regulator of the classical complement, is drastically up-regulated in FACS-sorted LPS-reactive murine astrocytes [11] and is also up-regulated in spinal cord lesions of progressive MS [5]. Taken together, the transcriptomic profile of EAE reactive astrocytes is consistent with a pro-inflammatory profile (overexpression of TSPO, serpinG1, and SPP1; low mRNA expression of arginase 1 and TGFβ, prototypic anti-inflammatory markers). This is in contrast to ischemia where cortical astrocytes exhibited a molecular phenotype suggesting that they may be protective [11]. We do not exclude that, in the context of autoimmune inflammation, white matter astrocytes re-express other molecules that would be beneficial as well. For example, increased expression of nuclear Estrogen Receptor α in reactive astrocytes has been shown to be instrumental for estrogen-mediated beneficial effects on EAE physiopathology [13, 42].

### Chemokines

It is well established that several chemokines are also expressed at low levels in resting astrocytes but are induced in a variety of inflammatory conditions. We looked at such prototypic chemokines whose expression has been shown to be induced in the EAE spinal cord tissue [3, 25, 26, 29, 43], in the MS brain [44–47], and in primary astrocyte cultures treated with pro-inflammatory cytokines [48–51]. Among tested chemokine transcripts, CCL2 (SCYA2/MCP-1), CCL5 (SCYA5/RANTES), and CCL7 (SCYA7/MCP-3), mRNA expressions were clearly induced in EAE samples (Table 3). Reactive astrocytes produce CCL2 in MS [46] as during EAE [13]. This expression is crucial for the recruitment of inflammatory monocytes and myelin-degrading macrophages [52]. This up-regulation seems restricted to the white matter astrocytes as it is not observed in gray matter during chronic EAE (Table S3 in [13]) as well as in MS [53]. Similarly, CCL7 is only detected in reactive astrocytes during EAE [42]. In line with these data, it has been shown that CCL2, 5, 7, CXCL9 (SCYB9), and CXCL10 (SCYB10/IP-10) expression are highly dependent on the inflammatory transcription factor NFkB and that transgenic mice with specific deletion of NFKB signaling in astrocytes exhibit decreased expression in those chemokines and reduced CNS inflammatory response during EAE [3, 4]. Thus, the high increase in these chemokine transcripts from our dissected astrocyte samples at the peak of EAE further validates our LCM strategy. In contrast, CXCL12 was very poorly detected in LCM-astrocytes, which is in agreement with the preferential CXCL12 expression by endothelial cells in this model [54], whereas in multiple sclerosis, CXCL12 is detected on blood vessels and astrocytes in chronic inactive and early active lesions [55]. Among other chemokine transcripts up-regulated during EAE, we also found CXCL16, whose expression and role in CNS injury is not well defined. CXCL16 transcript in contrast to previous chemokines was detected in control astrocytes and moderately increased in EAE samples (Table 3). CXCL16 is a transmembrane chemokine that can be cleaved to a soluble form by ADAM metalloproteinases and released by astrocytoma cells upon inflammatory conditions [56]. CXCL16 transcript was found up-regulated five-fold in spinal cord tissue in a slightly different murine EAE model [43]. Cultured astrocytes express CXCL16 [56]; recent data in MS reported astrocytic CXCL16 expression [57] and that serum levels of CXCL16 reflect disease activity [58]. The CXCL16 receptor, CXCR6, is expressed by activated T cells and neutrophiles [59] and is important, though not sufficient, for CNS T cell infiltration or motility [60]. Administration of CXCL16 antibody in adoptive transfer EAE reduced mononuclear cell trafficking and EAE symptoms [61]. However, recent data highlighted a neuroprotective role of CXCL16 in a murine model of pMCAO [62]. The mechanism of action involved astrocytic release of CCL2, which is rather deleterious for white matter during EAE as stressed above. Whether the increase in astrocytic CXCL16 expression is deleterious or protective during MS needs further investigation.

### Enzymes involved in steroid hormone inactivation

**Fig. 3:**
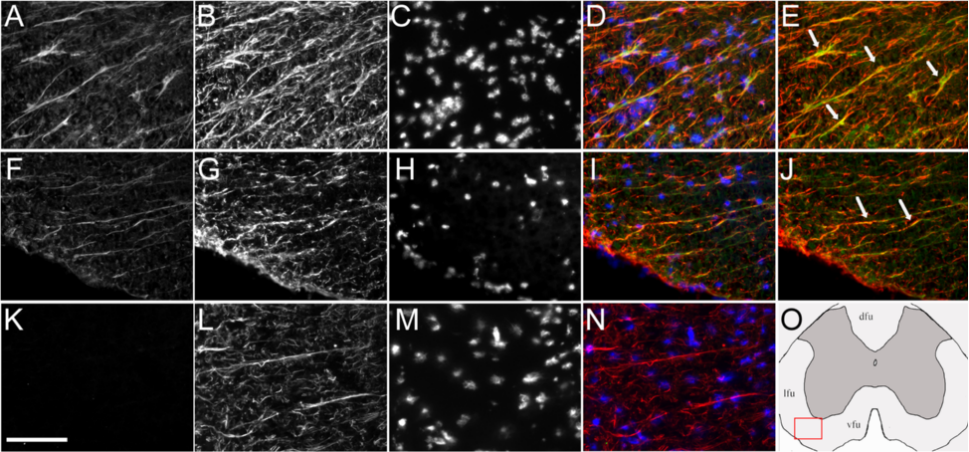
Astrocytic SULT1A immunoreactivity in the EAE and control white matter spinal cord. Representative stainings for EAE mouse (**a–e**) or control mouse (**f**–**j** and, for non-specific control, **k**–**n**). **a**, **f** SULT1A1 staining (*green channel*); **k** non-specific staining (no rabbit primary antibody, *green channel*); **b**, **g**, **l** GFAP staining (*red channel*); **c**, **h**, **m** DAPI staining (*blue channel*); **d**, **i**, **n** all channels merged; **e**, **j** red and green channels merged. **o** Schematic representation of the spinal cord section with approximate field (*red square*) used for the pictures. *Arrows* point to astrocytic (GFAP+) cells (with identified nucleus) in (**e**) or to astrocytic GFAP+ fibers in (**j**). **a**–**n** Scale bar, 75 μm

**Fig. 4:**
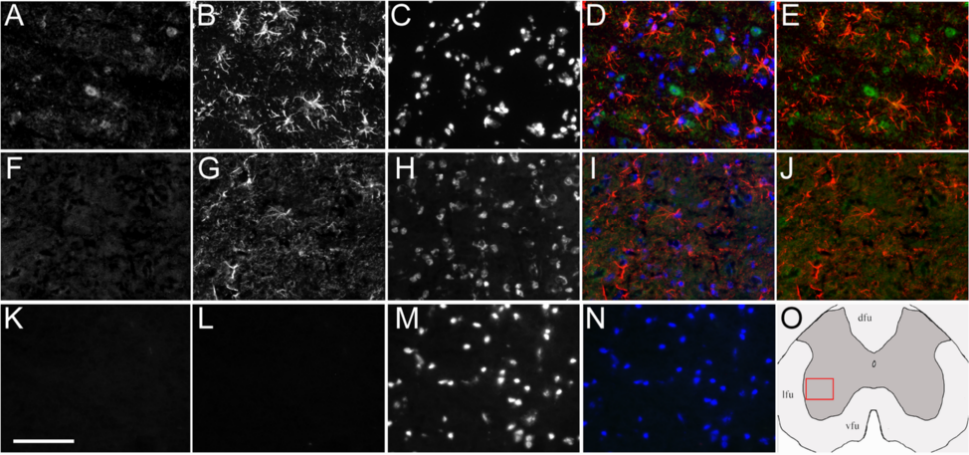
Lack of SULT1A1 immunoreactivity in astrocytes of the gray matter spinal cord. Representative stainings for EAE mouse (**a–e**) or control mouse (**f–j**), and for non-specific control (**k–n**). **a**, **f** SULT1A1 staining (*green channel*); **k**, **l** non-specific staining in absence of rabbit and rat primary antibodies (**k**, *green channel*; **l**, *red channel*); **b**, **g** GFAP staining (*red channel*); **c**, **h**, **m** DAPI staining (*blue channel*); **d**, **i**, **n** all channels merged; **e**, **j** red and green channel merged. **o** Schematic representation of the spinal cord section with approximate field (*red square*) used for the pictures. **a**–**n** Scale bar, 75 μm

We also looked for the expression of two classes of genes involved in toxin and steroid inactivation by glucuronidation (UGT enzymes) or sulfoconjugation (SULT enzymes). None of the glucuronosyltransferases tested were expressed in white matter LCM-astrocytes (Table 3), whereas they were easily detected in the mixed tissue control that contains liver, an organ rich in UGT and SULT enzymes. Among all sulfotransferases, SULT1E1 and SULT1A1 are the only enzymes having estrogen sulfonation activity. Sult1e1 mRNA is not expressed in neural tissue [14]. We could not detect it from mouse spinal cord cDNA (100 ng) by Real-Time PCR (data not shown) nor was it detected here in LCM-dissected astrocytes. We did not detect the expression of other sulfotransferases such as the androgen sulfotransferase Sult2a1 in LCM-dissected astrocytes. In contrast, relative good signals were obtained for Sult1a1 in control astrocytes, and the expression was further significantly increased in EAE astrocytes (Table 3). Single Real-Time PCR analysis of Sult1a1 transcripts from the whole spinal cord cDNA (100 ng) confirmed up-regulation at day postimmunization 16 (early EAE, 4.6 ± 0.1-fold) to day postimmunization 28 (late EAE, 4.9 ± 0.6) as compared to controls (*n* = 4/each group), indicating a sustained up-regulation of the mRNA during this chronic EAE model. Immunofluorescence performed on spinal cord sections from control or EAE mice confirmed the specific expression of SULT1A1 in GFAP+ astrocytes; SULT1A1 antibody effectively labeled cytoplasmic astrocytic fibers in the white matter (Fig. 3). The difference in protein expression was not as evident as for mRNA expression, but thick astrocytic processes and white matter astrocytes were more easily detected in EAE mice than in control mice in which most fine astrocytic process appeared weakly labeled (Fig. 3). Interestingly, we did not detect SULT1A1 immunoreactivity in astrocytes of the gray matter, where only some staining appeared restricted to a few neuronal nuclei (Fig. 4). Immunohistochemistry on paraformaldehyde-perfused tissue sections also identified similar pattern of immunoreactivity with both SULT1A1 antibodies: low staining of control astrocytic-like fibers and better visualization in EAE samples of astrocytic-like fibers in the white matter (Additional file 2: Figure S2). These data corroborate the transcript analysis at the protein level. Taken together, among the analyzed transcripts for sulfotransferases, the expression of SULT1A1 in normal and reactive white matter (WM) astrocytes is peculiar. Moreover, any of the UGT enzymes tested were found to be expressed in our astrocyte TLDA analysis, suggesting that white matter astrocytes are not well equipped to conjugate steroids and other substrates by glucuronidation. Interestingly, Sult1a1 mRNA was reported recently to be also induced in the spinal cord tissue of Lewis rat in a model of acute EAE [26], but its cellular origin was not determined. Moreover, when looking at microarrays that have been previously performed on MS tissue lesions, we found a report indicating that Sult1a1 transcript expression was two-fold increased in active lesions (three patients) but not in inactive lesions from another patient (Supplementary 1 in [6]). Because all four patients were analyzed for statistics, this up-regulation did not reach significance and was not highlighted. Nevertheless, this indicated that Sult1a1 transcript is expressed in human CNS tissue and particularly in active lesions that contains reactive astrocytes. Further experiments will determine whether astrocytes are involved as well in this up-regulation.

## Conclusion

We present here for the first time a selected transcriptomic (TLDA) profile from ex vivo LCM-WM astrocytes from adult mouse spinal cord in controls and during autoimmune neuroinflammation. Among the gene transcripts analyzed that established a molecular signature of WM reactive astrocytes, we highlighted here that SULT1A1, an enzyme having estrogen inactivating activity, is well expressed by the spinal cord WM astrocytes and further increased in EAE.

Whereas GFAP represents the best and widely used astrocytic marker, the volume covered by the GFAP staining is only 13 % of the volume of cortical protoplastic astrocytes [63], highlighting the need for additional astrocytic makers. Glutamate transporters GLAST-1 (EAAT1/ SLC1A3) and GLT-1 (EAAT2, SLC1A2) are often used to label and select resting astrocytes for in vitro studies or for transgenic mice [64, 65]. However, their expression is down-regulated in several EAE models [32, 66]. Moreover, these genes are also expressed in oligodendrocytes in human white matter [67] making them poor markers for identifying reactive astrocytes during neuroinflammation. Aldh1L1 is very specific to astrocytic populations but is drastically down-regulated in the mature spinal cord compared to brain regions [65]. In fact, in mouse brain gray matter, Sult1a1 transcript is highly enriched in S100 promoter-driven GFP+ FACS-sorted P16-17 astrocytes [10] as well as in adult Aldh1L1 expressing astrocytes [68]. Sult1a1 may thus be used as an additional astrocytic marker for immunocytochemistry or tracing studies in adult mouse. Our data indicate that, at the protein level detected by conventional immunocytochemistry or immunofluorescence, SULT1A1 in the spinal cord is preferentially expressed in white matter astrocytes (ventral, lateral and dorsal funiculi) as compared to gray matter astrocytes. Similarly, we were unable to detect SULT1A1 immunoreactivity in mouse cortical gray matter astrocytes, whereas SULT1A1-immunoreactive astrocytes could be detected in the white matter tracts of the forebrain (data not shown). Since SULT1A1 staining was less intense as compared to GFAP, we cannot conclude whether only a subpopulation of WM astrocytes expresses SULT1A1. Further experiments at the single cell level would be needed to address this point.

Recent studies have focused on radial glia and astrocytes as important steroid synthetic cells that provide active neurosteroids for local neuroendocrine functions and regulation of neurogenesis [69]. On the other hand, studies on CNS tissues have highlighted decreases in the expression of enzymes involved in neurosteroid biosynthesis in EAE [26] or MS [70]. In light of our observations, as well as the reported Sult1a1 transcript expression in MS active lesions [6] and the estrogen sulfonation activity of SULT1A1, reactive astrocytes may have not only altered sex steroid synthesis but also higher estrogen conjugation activity, both potentially leading to lower intracellular concentrations active estrogen. Strikingly, white matter reactive astrocytes express nuclear estrogen receptor alpha (ESR1) during EAE [13] as well as in MS active lesions [38, 71], so they are well equipped to respond to low doses of estrogen. However, only high nanomolar estrogen concentrations are effective in reducing ongoing EAE and chemokine expression such as CCL2 [13]. Interestingly, 17α-ethinylestradiol (EE2), an estrogen commonly used in oral contraceptives, acts as an inhibitor of SULT1A1 but not as a substrate [72]. This may account for the effectiveness of this compound when administrated after disease onset to reduce murine EAE [73], whereas only high nanomolar doses of the natural ligand 17β-estradiol are effective [13]. The astrocytic expression of SULT1A1, which is further increased during EAE, may account for the relative resistance of this cell population to respond to exogenous low doses of 17β-estradiol. This may hold true as well for MS astrocytes, an issue which now requires further investigation because a recent MS clinical trial of a 17β-estradiol-based protocol starting at postpartum—to prevent the increased risk of MS relapse observed at this period—has been recently disappointing [74]. Finally, in human, *Sult1a1* polymorphisms [75] and copy number variations [76] associated with differences in sulfonation activity have been reported. Thus, large-scale genome-wide association studies may also consider examining *Sult1a1* gene as previously highlighted [77]. This could well apply to MS genomic studies.

## Additional files

Additional file 1: Figure S1. FACS analysis of EAE spinal cords used for LCM. The lymphocytic infiltrates were composed mostly of CD4+ effector/memory T cells and macrophages. A-E, Flow cytometric scattergrams of a representative spinal cord cell suspension in the presence of myelin debris. A, Side scatter showing the gating of leukocytes stained with DAPI and excluding myelin debris. B, Example of CD45 and CD11b labelings on DAPI+ gated cells. Microglia are in green (CD45int CD11b+), macrophages (CD45high CD11b + Ly6G-) and neutrophiles (CD45high CD11b + Ly6G+) in yellow, and lymphocytes (CD45high CD11b-) in purple. C, Example of CD4 and CD8 labelings on CD3+ CD45high DAPI+ gated cells. D, Example of CD19 and B220 labelings on CD45high DAPI gated cells. B220 (Q1 + Q2) was used to select B cells in the lymphocyte population as CD19 antibody gave poor signal (in contrast to a spleen cell control, data not shown). E, Example of CD44 and CD62L labeling on CD45high CD3+ DAPI+ gated cells. F, Corresponding results obtained for CD4+ and CD8+ T cells with naive (CD44- CD62L+), central memory (CD44 + CD62L+) and effector/memory (CD44 + CD62L-) phenotypes from the three mouse spinal cords (mean ± sem). G. Summary of leukocyte populations (% of all CD45high cells) in the three mouse EAE spinal cords (mean ± sem). H, Summary of lymphocyte composition (% of CD45high CD11b- cells) in the three mouse EAE spinal cords (mean ± sem). Additional file 2: Figure S2. Immunohistochemistry for SULT1A1 in the white matter spinal cord. DAB/peroxidase immunohistochemistry was applied to detect SULT1A1 in sections from paraformaldehyde-perfused female mice, using either Abcam or Bioss rabbit antibody as indicated for each picture on the top left. The tissue that was used (control or EAE) is indicated on the top right. SULT1A1 immunoreactivity is detected in astrocyte/radial glia- like fibers of the white matter (vfu, ventral funiculus, dfu, dorsal funiculus), especially on EAE samples. The pictures result from identical time of acquisition. Adjacent sections were used to check for GFAP staining using a rabbit antibody in corresponding areas. As positive control, SULT1A1-immunoreactivity was detected in kidney tubules with the two antibody tested. For the picture of the kidney assessing the lack of non-specific (NS) staining -when no primary antibody was included in the first incubation step-, differential interference contrast (DIC)/Nomarski interference was added to see the unlabeled tubules. Scale bar, 60 μm for spinal cord (SPC) or 100 μm for kidney.
