# Supplementary figures and images for: Transcript analysis of laser capture microdissected white matter astrocytes and higher phenol sulfotransferase 1A1 expression during autoimmune neuroinflammation
Source: J Neuroinflammation. 2015 Jul 4;12:130. doi: 10.1186/s12974-015-0348-y (PMC4501186; doi:10.1186/s12974-015-0348-y)

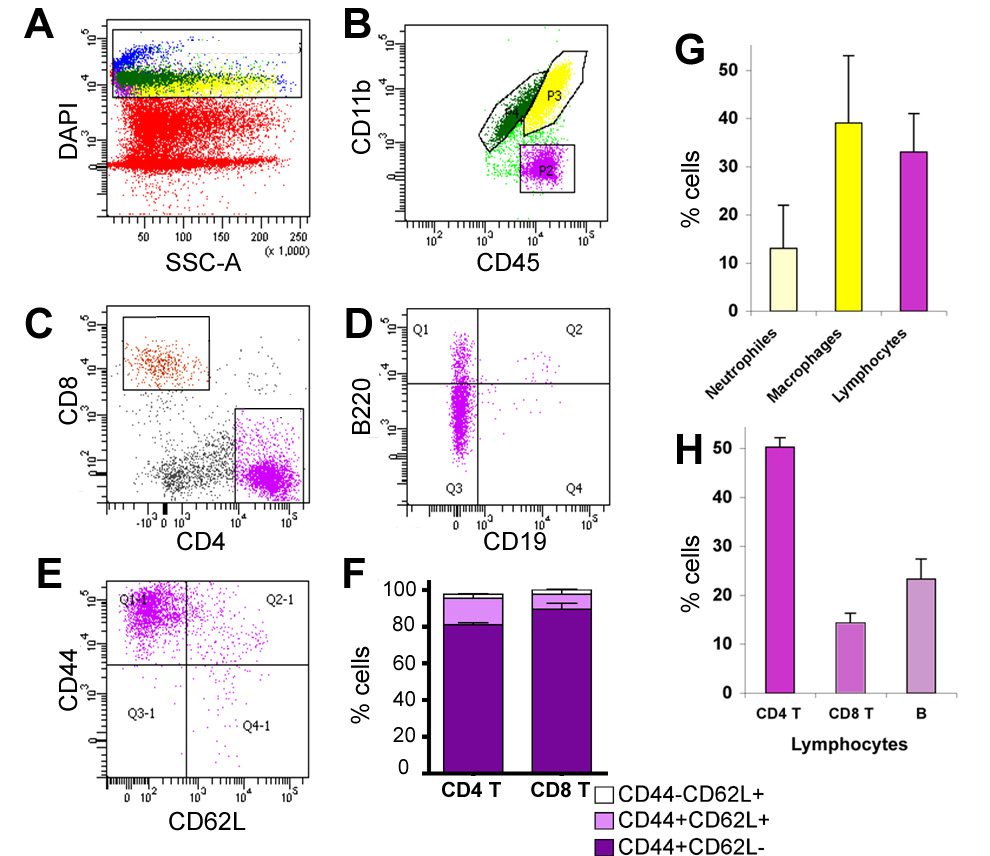

Supplement: Additional file 1: Figure S1. — FACS analysis of EAE spinal cords used for LCM. The lymphocytic infiltrates were composed mostly of CD4+ effector/memory T cells and macrophages. A-E, Flow cytometric scattergrams of a representative spinal cord cell suspension in the presence of myelin debris. A, Side scatter showing the gating of leukocytes stained with DAPI and excluding myelin debris. B, Example of CD45 and CD11b labelings on DAPI+ gated cells. Microglia are in green (CD45int CD11b+), macrophages (CD45high CD11b + Ly6G-) and neutrophiles (CD45high CD11b + Ly6G+) in yellow, and lymphocytes (CD45high CD11b-) in purple. C, Example of CD4 and CD8 labelings on CD3+ CD45high DAPI+ gated cells. D, Example of CD19 and B220 labelings on CD45high DAPI gated cells. B220 (Q1 + Q2) was used to select B cells in the lymphocyte population as CD19 antibody gave poor signal (in contrast to a spleen cell control, data not shown). E, Example of CD44 and CD62L labeling on CD45high CD3+ DAPI+ gated cells. F, Corresponding results obtained for CD4+ and CD8+ T cells with naive (CD44- CD62L+), central memory (CD44 + CD62L+) and effector/memory (CD44 + CD62L-) phenotypes from the three mouse spinal cords (mean ± sem). G. Summary of leukocyte populations (% of all CD45high cells) in the three mouse EAE spinal cords (mean ± sem). H, Summary of lymphocyte composition (% of CD45high CD11b- cells) in the three mouse EAE spinal cords (mean ± sem). [file 12974_2015_348_MOESM1_ESM.jpg]

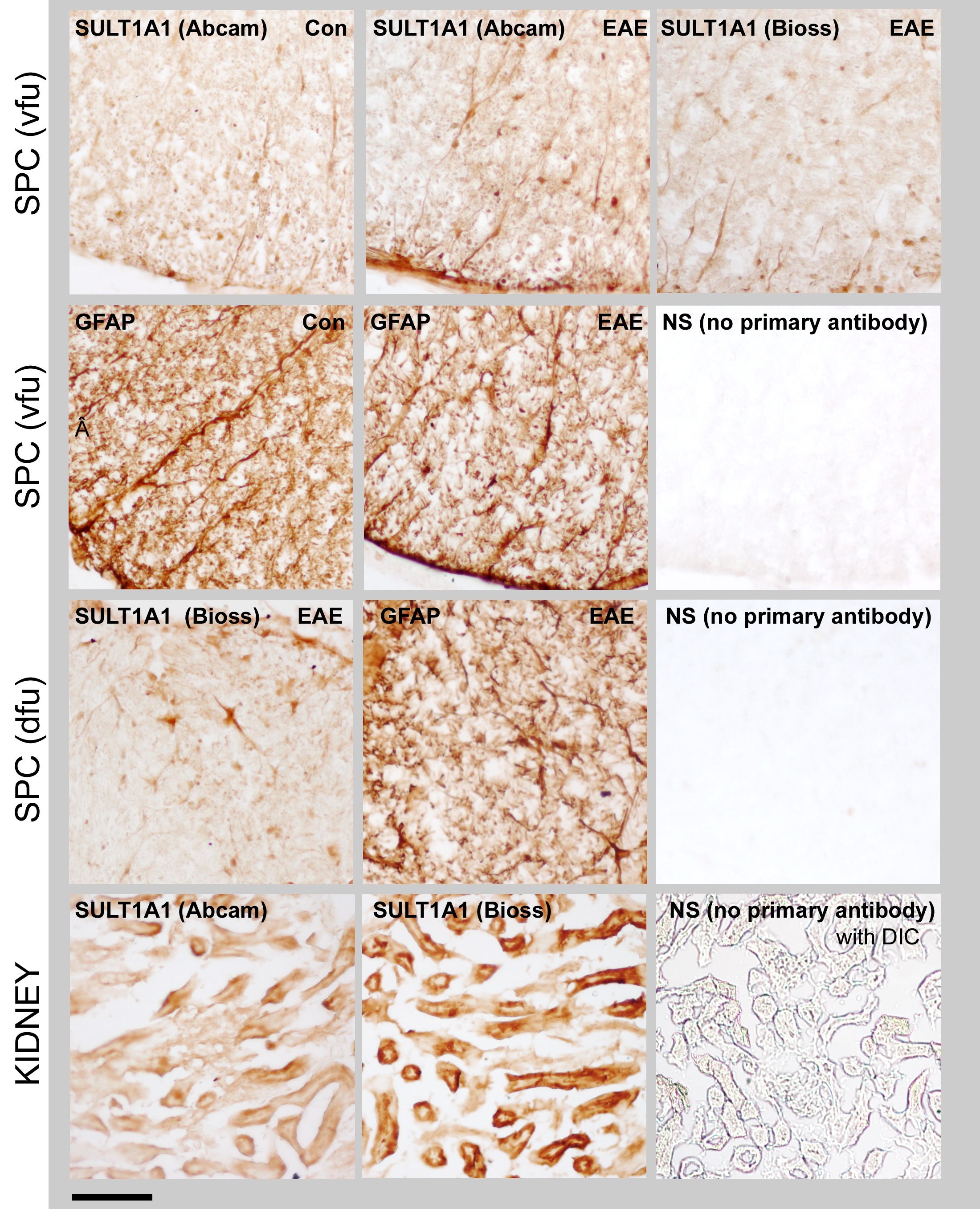

Supplement: Additional file 2: Figure S2. — Immunohistochemistry for SULT1A1 in the white matter spinal cord. DAB/peroxidase immunohistochemistry was applied to detect SULT1A1 in sections from paraformaldehyde-perfused female mice, using either Abcam or Bioss rabbit antibody as indicated for each picture on the top left. The tissue that was used (control or EAE) is indicated on the top right. SULT1A1 immunoreactivity is detected in astrocyte/radial glia- like fibers of the white matter (vfu, ventral funiculus, dfu, dorsal funiculus), especially on EAE samples. The pictures result from identical time of acquisition. Adjacent sections were used to check for GFAP staining using a rabbit antibody in corresponding areas. As positive control, SULT1A1-immunoreactivity was detected in kidney tubules with the two antibody tested. For the picture of the kidney assessing the lack of non-specific (NS) staining -when no primary antibody was included in the first incubation step-, differential interference contrast (DIC)/Nomarski interference was added to see the unlabeled tubules. Scale bar, 60 μm for spinal cord (SPC) or 100 μm for kidney. [file 12974_2015_348_MOESM2_ESM.jpg]
